# Inhibitor of differentiation 3 confers the robust anti‐tumor activity of Kupffer cells

**DOI:** 10.1002/mco2.708

**Published:** 2024-08-31

**Authors:** Jiang Ren, Sijia Liu, Long Zhang

**Affiliations:** ^1^ The First Affiliated Hospital, MOE Basic Research and Innovation Center for the Targeted Therapeutics of Solid Tumors Institute of Biomedical Innovation, School of Basic Medical Sciences, Jiangxi Medical College, Nanchang University Nanchang China; ^2^ International Biomed‐X Research Center, Key Laboratory of Precision Diagnosis and Treatment for Hepatobiliary and Pancreatic Tumor of Zhejiang Province, Second Affiliated Hospital of Zhejiang University School of Medicine, Zhejiang University Hangzhou China; ^3^ MOE Key Laboratory of Biosystems Homeostasis & Protection and Innovation Center for Cell Signaling Network, Life Sciences Institute, Zhejiang University Hangzhou China

1

A recent landmark study published in *Nature* by Deng et al. reveals that inhibitor of differentiation 3 (ID3) empowers Kupffer cells (KCs) to efficiently engulf live tumor cells and activate the lymphoid anti‐tumor immune response, orchestrating a potent anti‐tumor niche and restricting liver tumor growth (Figure 1).^1^ The intricate interplay between macrophages and cancer has been the subject of intense research in recent decades. This study represents a milestone in the field of cell therapy, providing novel insights into tumor dynamics in an organ‐specific context and offering promising prospects for the future application of engineered ID3‐expressing macrophages in cancer immunotherapy.

**FIGURE 1:**
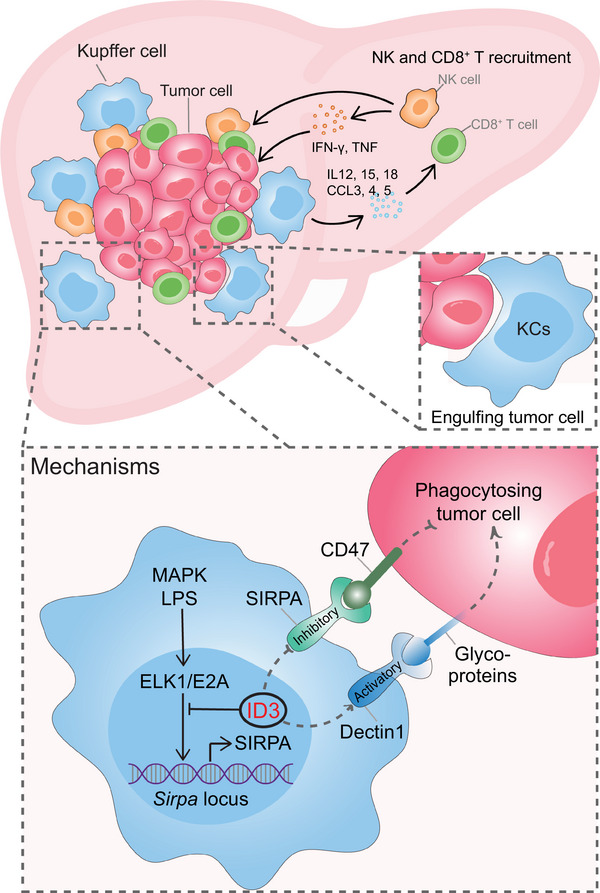
Inhibitor of differentiation 3 (ID3)‐dependent anti‐tumor immunity of liver‐resident Kupffer cells (KCs). KCs are surrounded by liver metastasis tumor nodules, forming a robust anti‐tumor immune niche. Mechanistically, ID3 prevents the binding of transcription factors ELK1 and E2A to the *Sirpa* locus, resulting in decreased expression of signal regulatory protein α (SIRPA) and increased expression of dendritic cell‐associated C‐type lectin‐1 (Dectin1) in adult KCs. As a result, The CD47–SIRPA inhibitory signaling is limited, while the Glyco‐antigen–Dectin1 activation signaling is augmented. Activated KCs can directly and continuously engulf and kill live tumor cells. Additionally, KC activation triggers the secretion of chemokines (CCL3, 4, and 5) and interleukins (IL12, IL15, and IL18), recruiting and activating NK cells and CD8^+^ T cells to produce anti‐tumor cytokines (IFNγ and TNF) for eliminating tumor cells.

The macrophage population comprises cells derived from diverse lineages that undergo differentiation during embryonic development, with KCs predominantly representing hepatic resident macrophages.^2^ The authors initially employed the CSF1R inhibitor PLX5622 or the genetic tool *Clec4f* ^cre^ *Csf1r* ^f/f^/*Spi1* ^f/f^ to deplete KCs in tumor‐bearing mice, and the results demonstrated a significant increase in liver metastatic tumor cells in KC‐deficient mice. The spatial distributions of macrophages within the tumor microenvironment (TME) of liver metastases exhibit substantial heterogeneity. Tumor‐associated macrophages (F4/80^+^TIM4^−^) are clustered within the tumor nodules, while KCs (TIM4^+^CLEC4F^+^) form a peritumoral niche consistently surrounding the tumor nodules.

The subsequent RNA‐seq analysis of sorted KCs isolated from tumor‐bearing livers revealed an upregulated expression of various receptors associated with the activation of macrophages and effector lymphoid cells, including activating receptors such as dectins, chemotactic factors like CCL2/3/4/5/6/7, and interleukins like IL‐12/15/18. The authors observed a high abundance of KCs in tumor‐derived material in both short‐ and long‐term orthotopic models, as well as in endogenous KPC tumor models with spontaneous metastasis. The direct engulfment and killing of live tumor cells by KCs were demonstrated through in vitro and in vivo live‐cell time‐lapse imaging. Immunofluorescence staining experiments conducted on tumor‐bearing livers have substantiated the production of CCR5 ligands (CCL3/4/5) and cytokines (IL‐12/15/18) by KCs at the periphery of tumors. Additionally, the authors observed preferential enrichment of activated natural killer (NK) cells and CD8^+^ T cells at the tumor margin in the metastatic liver. This was accompanied by KCs expressing CCL3, CCL4, CCL5, IL‐12, IL‐15, and IL‐18, which may contribute to enhanced phagocytosis of tumor cells.

KCs serve as resident immune sentinels and are regarded as a robust immune barrier against tumor progression owing to their high phagocytic capacity, however, the underlying mechanism remains largely elusive. ID3 protein has been demonstrated to function as a pivotal nuclear factor governing the lineage of KCs. The deficiency of *Id3* impedes the differentiation process of KCs in mouse embryos, leading to a reduction in the quantity of KCs.^3^ The authors evaluated the consequences of *Id3* deletion in adult KCs in *Clec4f* ^cre^ *Id3* ^f/f^ mice, which enables conditional deletion of ID3 specifically in adult KCs without impairing their numbers, morphology, and particle uptake ability. Results displayed that the deletion of *Id3* in adult KCs resulted in the development of extensive liver metastases and a shortened survival time in comparison to the controls. The findings suggested that the presence of ID3 is essential for the anti‐tumor activity exhibited by adult KCs. Differential gene expression analysis of *Id3*‐deficient KCs and intact KCs revealed that pathways associated with signaling receptor activity, leukocyte‐mediated cytotoxicity, leukocyte migration, and T cell‐mediated immunity exhibited downregulation in *Id3*‐deficient KCs. The authors discovered that the deficiency of *Id3* skews the balance of activating/inhibitory receptors towards an upregulation of inhibitory receptor expression. This subtle modulation plays a crucial role in enabling KCs to establish a potent anti‐tumor immune environment.

The expression of ID3 is higher in KCs compared to other macrophage subsets, including microglia, alveolar macrophages, and bone marrow‐derived macrophages (BMDMs), whereas the levels of signal regulatory protein‐α (SIRPA) are comparatively lower. CD47, a ligand of SIRPA, predominantly expresses on tumor cells. Upon binding, SIRPA triggers a signaling cascade that results in the suppression of phagocytosis.^4^ In contrast, the dendritic cell‐associated C‐type lectin‐1 (Dectin1) expressed on macrophages recognizes tumor cell antigens, thereby activating macrophages within tumors and facilitating the priming cytotoxic T‐cell responses.^5^ Blockade of SIRPA rescues the expression of Dectin1/chemokines/cytokines by ID3‐ deficient KCs, thereby restoring the peritumoral anti‐tumor niche mediated by KCs, including the recruitment and activation of CD8^+^ T and NK cells.

The liver continuously receives venous blood from the gastrointestinal tract, thereby exposing the KCs to circulating tumor cells and microbial products such as lipopolysaccharide (LPS). Molecular‐level analysis showed that ID3 effectively hindered the interaction between transcription factors E2A and ELK1 with the *Sirpa* promoter, as well as the upstream and intronic enhancer regions of *Sirpa* in KCs. Activation of the ELK1/E2A‐SIRPA pathway through LPS or MAPK signaling can be counteracted by ID3. Additionally, the ID3‐dependent anti‐tumor ability of KCs can be extended to other macrophages. Ectopic expression of mouse *Id3* in mouse BMDMs or human *ID3* in human induced pluripotent stem cell‐derived macrophages exhibited augmented phagocytic activity and heightened production of inflammatory cytokines in vitro. The injection of ID3‐overexpressing BMDMs into mouse tumor models resulted in a significant inhibition of tumor growth and metastasis, accompanied by enhanced recruitment and activation of NK and CD8^+^ T cells across multiple mice tumor models.

In conclusion, this study elegantly elucidates that ID3 plays a pivotal role in enhancing the anti‐tumor immune response mediated by KCs through buffering SIRPA transactivation in the liver. In addition to SIRPA, ID3 may also modulate the expression of other genes and influence signaling pathways. Therefore, revealing the landscape of the ID3 regulatory network is imperative for a comprehensive understanding of its role in immune regulation. ID3 represents a prominent target gene of bone morphogenetic protein (BMP) signaling, thereby highlighting the potential for exploiting BMP signaling agonists to enhance the efficacy of tumor immunotherapy. Prospectively, this study also illuminates the potential of harnessing ID3 expression in engineering macrophages to augment the efficacy of existing therapies or develop novel immunotherapy strategies. However, the TME displays remarkable heterogeneity, encompassing a diverse array of cellular components and intricate signaling molecules. Therefore, it is crucial to further investigate the preclinical evaluation of ID3‐expressing macrophages within the TME in order to comprehensively understand their functional efficacy and potential side effects in tumor immunotherapy.

## AUTHOR CONTRIBUTIONS

J.R. and S.L. conceived and drafted the manuscript. L.Z. provided valuable discussion. All authors have read and approved the article.

## CONFLICT OF INTEREST STATEMENT

The authors declare no conflict of interest.

## ETHICS STATEMENT

Not applicable.

## Data Availability

Not applicable.
